# Orphan medical devices have come a long way

**DOI:** 10.1186/s13023-023-02685-7

**Published:** 2023-04-05

**Authors:** M. Dooms

**Affiliations:** IRDiRC Working Group on MedTech for Rare Diseases, University Hospitals Leuven, Leuven, Belgium

**Keywords:** Rare diseases, History of medicine, Humanitarian-use device, Custom-made medical device, Medtech, Repurposing

## Abstract

**Background:**

In many countries worldwide orphan drug regulations are installed but only the United States of America and Japan have an orphan device regulation. For many years surgeons have used off-label or self-assembled medical devices for the prevention, diagnosis or treatment of rare disorders. Four examples are given: an external cardiac pacemaker, a metal brace for clubfoot in newborns, a transcutaneous nerve stimulator and a cystic fibrosis mist tent.

**Conclusion:**

In this article we argue that we need authorized medical devices as well as medicinal products to prevent, diagnose and treat patients with life-threatening or chronically debilitating disorders with a low prevalence/incidence. Several arguments are given to support this statement.

## Main text

Orphan medical devices are products or equipment intended for the prediction, prevention, diagnosis, support, treatment and management of life-threatening or chronically debilitating diseases with a low prevalence/incidence. Orphan medical technology is then considered as both the device (tool/equipment) and the connectivity of the device.

Previous, very limited data have shown that there are few devices on the market specific for these indications, but both medical specialists and patients have strongly indicated at many occasions the need for more and specific orphan medical devices [1]. In a US survey (there is no EU data available), this number was as high as over 90% of medical specialists indicating the need for one or more devices, indicating indeed a very strong need for more orphan devices being developed. The recent Medical Device Regulation in Europe does not even mention this unmet need. Similar as to orphan drugs, most devices start their development in an academic, collaborative setting or under off-label use, so making this a very suitable topic for research.

We can mention numerous examples, but some examples are:

- An adapting prosthesis after rare cancer pediatric surgery: as the child grows, the device needs to grow with the child
- An exoskeleton, to support rare disease patients that have muscle weakness
- Implantable pacemakers for children with rare heart conditions
- Smartphone apps that help support parents with their child’s nutrition and drug regimen
- A diaphragm support as to help patients continue breathing
- A device alarming small and big seizures in rare epilepsy conditions.

Similar to orphan drugs, there is much research and development needed in this area, incentives to stimulate authorization and reimbursement, tools for patient involvement and materiovigilance.

## Historical background

In a previous article [1] I described some historical examples of medical devices used in the prevention, diagnosis and treatment of rare diseases: negative pressure ventilator, stereo-tactic brain surgery “robot”, incubator, dialyzer and orthopedic traction table. In this article I will present more historical orphan devices and highlight the still high need for a legislation to authorized such devices.

### External cardiac pacemaker

**Fig. 1:**
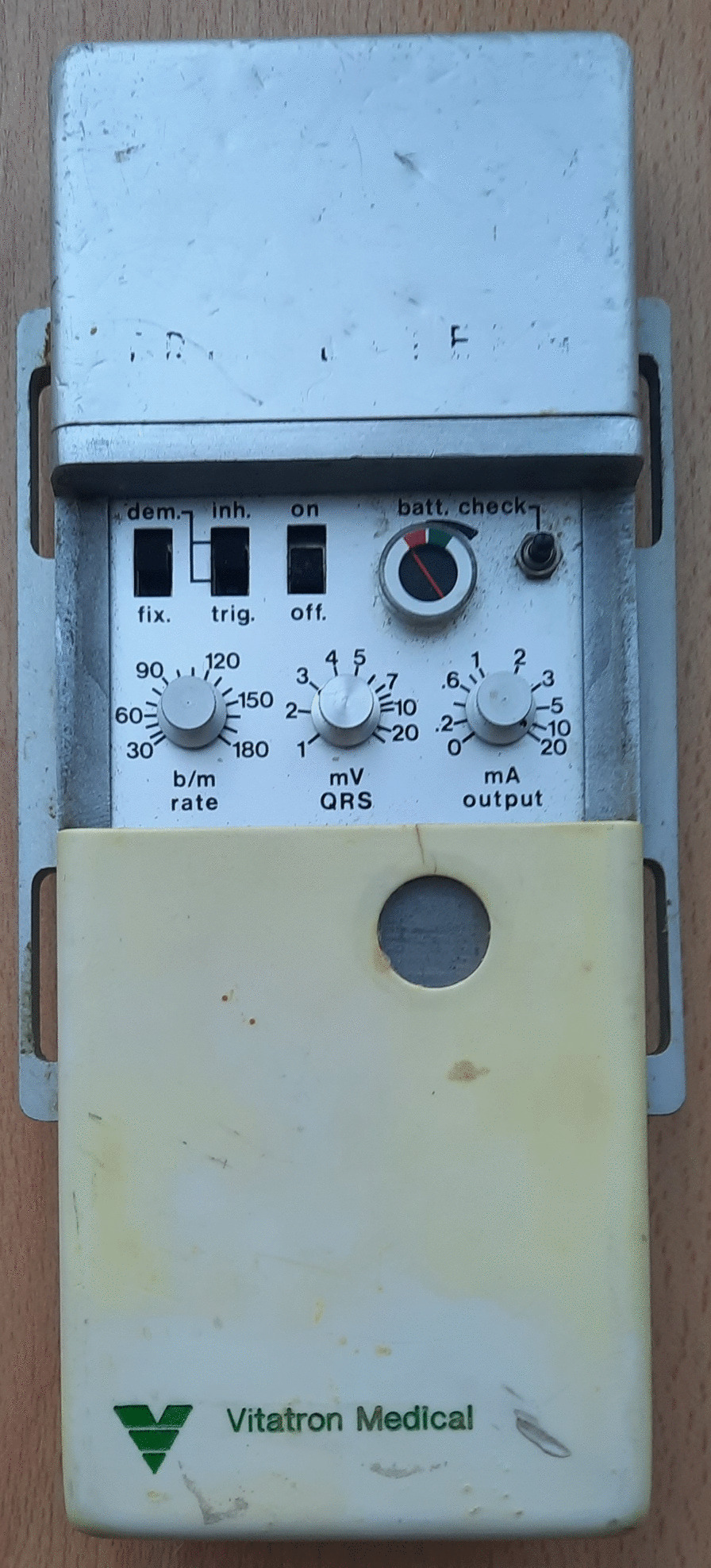
External cardiac pacemaker

The external cardiac (“trans cutaneous” or “artificial”) pacemaker is an electrodes based medical device used to regulate the contractility of myocardiocytes to maintain heart rate. For example the external Vitatron Medical pacemaker (14 cm–9 cm–2.5 cm) with 9 V battery produces pulses of 1 ms (Fig. 1). The first implantable pacemaker was produced by Rune Elmqvist and implanted in the Karolinska Hospital in Sweden by Ake Senning in 1958. Thanks to miniaturization it can now be implanted in children with rare cardiac disorders with excellent clinical results.

### Metal brace for clubfoot in newborns

**Fig. 2:**
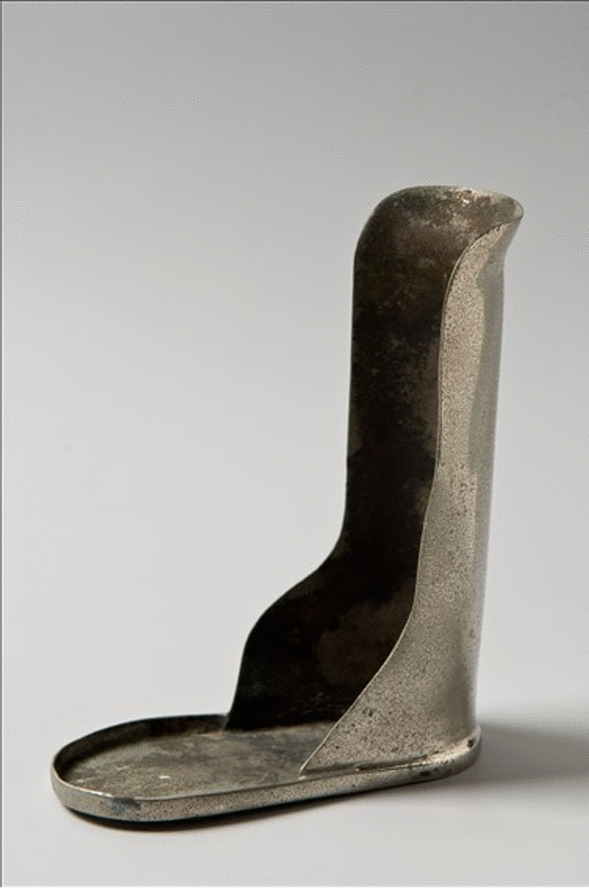
Metal brace for clubfoot

Clubfoot or congenital talipes equinovarus (CTEV) is a birth defect where one or both feet are rotated inward and downward. Genetic as well as environmental factors may be involved. Diagnosis may be made during an ultrasound (usual onset during early pregnancy) or at birth. Treatment with good prognosis involves moving the foot into an improved position followed by casting, repeated weekly. In the older days a metal brace was used (Fig. 2). Today initial casting during approximately 6 weeks followed by a surgical procedure and continued casting for several weeks with finally brace or splint support will lead to total cure.

### Transcutaneous electrical nerve stimulator

**Fig. 3:**
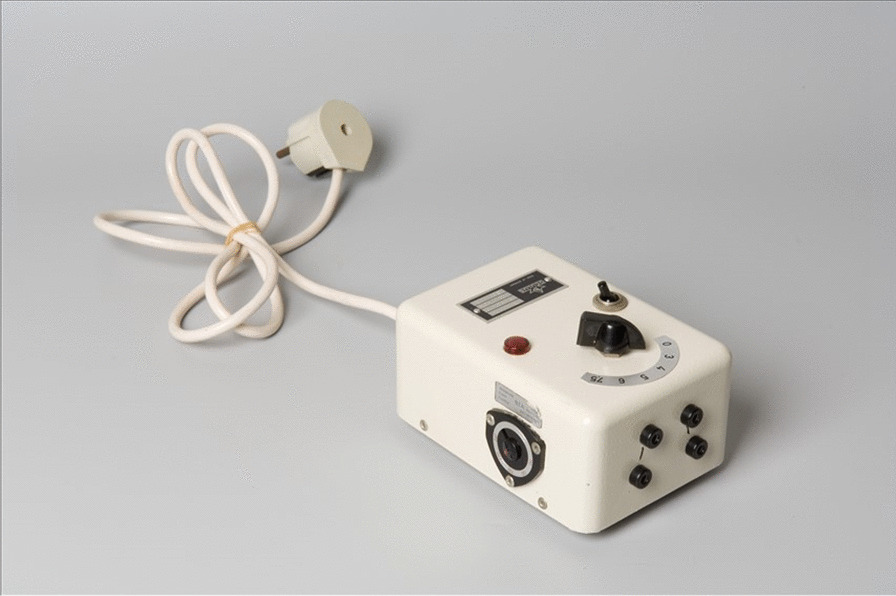
External trans-cutaneous nerve stimulator

Neurostimulation is the purposeful stimulation of the nervous system by invasive (micro-electrodes) or non-invasive means. This device can improve the quality of life of rare disease patients with profound loses to various sense organs such as dystonia. Peripheral nerve stimulation has a long history [2]. An early device made by Oculus, Germany (Fig. 3) with a 0.1 A fuse could produce electrical pulses between 0 and 7.5 V to 4 electrodes. A wide variety of innovative devices for neuromodulation are available today.

### Cystic fibrosis mist tent therapy

**Fig. 4:**
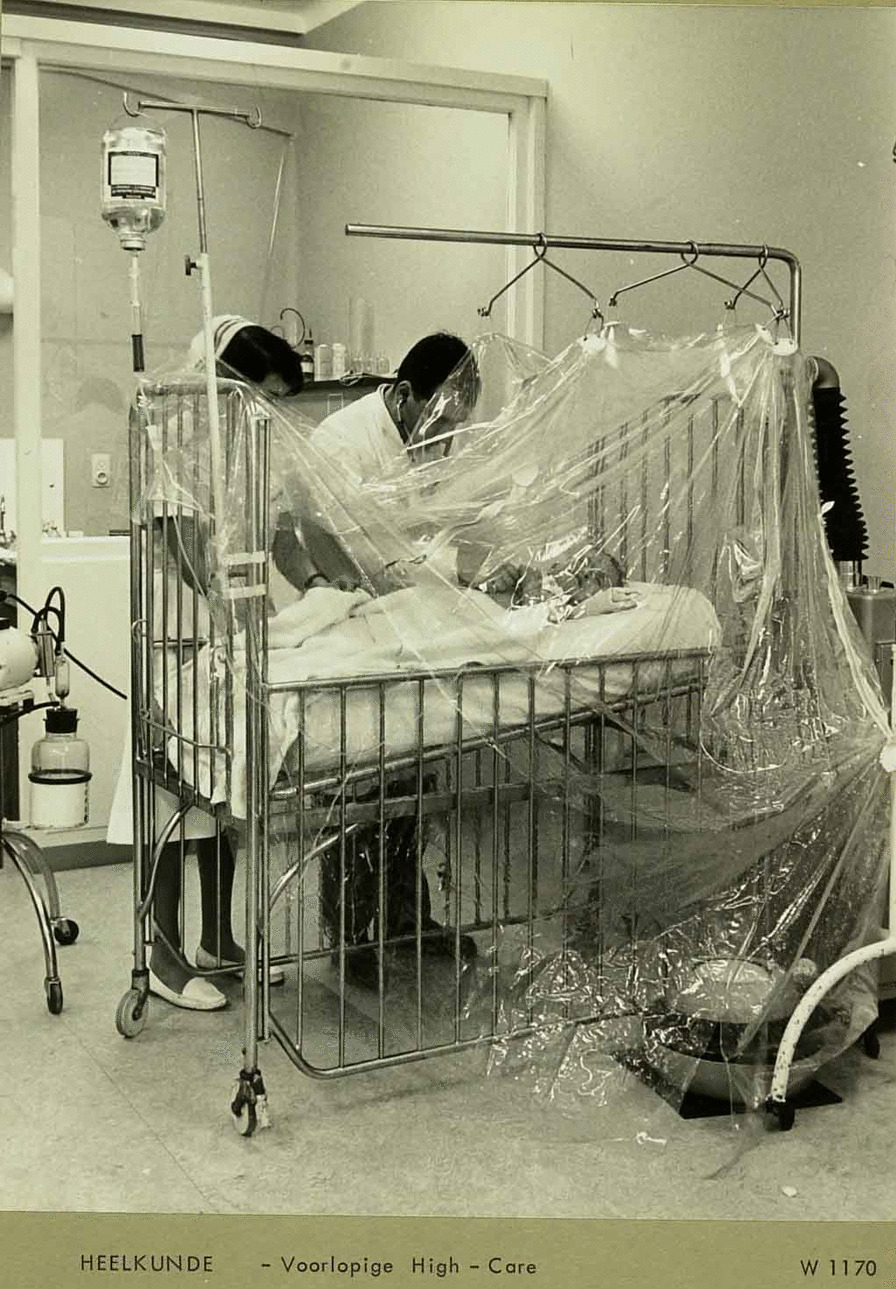
Cystic fibrosis mist tent therapy

A mist tent (also called croupette or cool-humidity tent) houses a nebulizer that transforms distilled water into mist (Fig. 4). This device eased breathing and helped to decrease respiratory tract excretions [3]. Oxygen through a nasal tube and/or medications (e g Mesna) may be added and aspiration was performed. Bed sheets and blankets became wet and the child turned cold and blue overnight. Today new nebulizer s are widely available.

### Present situation

Today in 92 of the 194 countries worldwide, orphan drug regulatory measures are installed and only 2 countries have orphan device authorizations:

*As mentioned in the previous article [1] the Food and Drug Administration (FDA) has his “Humanitarian Use Device” Regulation since 1990 [4]. Recently the FDA has developed a Pediatric Medical Device Development plan [5] to understand and define optimization of innovation in devices for pediatric use.

*The Pharmaceuticals and Medical Devices Agency (PMDA) is the Japanese government organization in charge of reviewing drugs and medical devices, overseeing post-market safety and providing relief for adverse health effects [6]. The current PMDA has established in 2004 the Pharmaceuticals and Medical Devices Evaluation Center of the National Institute of Health Sciences (PMDEC).

*The new Medical Device Regulation 745/2017 in Europe did not mention medical devices for indications with a low prevalence. With the current implementation of this EU MDR, it has become even more difficult to place custom made devices on the market, including 3-D printed devices.

There also seems to be a big difference in the definition of a rare disorder if it concerns medicinal products or if it concerns devices. According to the FDA definition, an orphan drug is intended to treat a condition affecting fewer than 200 000 persons in the United States (332.403.650 inhabitants on 1 Jan 2022) and an orphan device (“Humanitarian Use Device”) is designed to treat or diagnose a disease or condition that affects or is manifested in not more than 4000 individuals in the United States per year. A uniform definition would help in this matter.

Business models (with incentives such as for orphan medicinal products) need to be developed to bring these devices to the market, stimulate the development of better devices (supportive frameworks), avoid withdrawal for financial reasons [7, 8], regulate off-label use and manage materiovigilance. Evidence may be established to work together in public private partnerships as a significant part of the the orphan device industry are small and medium sized enterprises, focused on single products.

Off-label use of medicinal products for rare disorders has lead to several authorizations of orphan medicinal products. Can we also perform repurposing for medical devices? Different from medicinal products, medical devices need continuous refinement (hard- and software) and have several incremental patents and products.

## Conclusion

We need orphan medical device regulations to authorize devices to prevent, diagnose or treat life-threatening or chronically debilitating disorders with a low prevalence/incidence as we have orphan drug regulations today.

All pictures are taken from the archives of HistArUz, the Museum of the History of Medicine and Pharmacy at the University Hospitals Leuven, Belgium: http://www.uzleuven.be/histaruz.

## Data Availability

Data sharing not applicable to this article as no datasets were generated or analysed during the current study.

## Abbreviations

CTEV: Congenital equinovavarus
EU: European Union
FDA: Food and drug administration
IRDiRC: International rare disease research consortium
MDR: Medical device regulation
PMDA: Pharmaceutical medical device agency (Japan)
PMDEC: Pharmaceutical and medical devices evaluation center (Japan)
US: United States of America
